# Application of Exogenous Silicon for Alleviating Photosynthetic Inhibition in Tomato Seedlings under Low−Calcium Stress

**DOI:** 10.3390/ijms232113526

**Published:** 2022-11-04

**Authors:** Zhaozhuang Li, Zeci Liu, Zhibin Yue, Jie Wang, Li Jin, Zhiqi Xu, Ning Jin, Bo Zhang, Jian Lyu, Jihua Yu

**Affiliations:** 1 College of Horticulture, Gansu Agricultural University, Lanzhou 730070, China; 2 Key Laboratory of Crop Science in Arid Environment of Gansu Province, Lanzhou 730070, China

**Keywords:** tomato, silicon, low−calcium stress, photosynthetic enzyme

## Abstract

To address the low Ca−induced growth inhibition of tomato plants, the mitigation effect of exogenous Si on tomato seedlings under low−Ca stress was investigated using different application methods. We specifically analyzed the effects of root application or foliar spraying of 1 mM Si on growth conditions, leaf photosynthetic properties, stomatal status, chlorophyll content, chlorophyll fluorescence, ATP activity and content, Calvin cycle−related enzymatic activity, and gene expression in tomato seedlings under low vs. adequate calcium conditions. We found that the low−Ca environment significantly affected (reduced) these parameters, resulting in growth limitation. Surprisingly, the application of 1 mM Si significantly increased plant height, stem diameter, and biomass accumulation, protected photosynthetic pigments, improved gas exchange, promoted ATP production, enhanced the activity of Calvin cycle key enzymes and expression of related genes, and ensured efficient photosynthesis to occur in plants under low−Ca conditions. Interestingly, when the same amount of Si was applied, the beneficial effects of Si were more pronounced under low−Ca conditions that under adequate Ca. We speculate that Si might promote the absorption and transport of calcium in plants. The effects of Si also differed depending on the application method; foliar spraying was better in alleviating photosynthetic inhibition in plants under low−Ca stress, whereas root application of Si significantly promoted root growth and development. Enhancing the photosynthetic capacity by foliar Si application is an effective strategy for ameliorating the growth inhibition of plants under low−Ca stress.

## 1. Introduction

Silicon, the second most abundant element on earth, is not essential for plant growth [1,2]; however, its unique role is irreplaceable by other elements [3,4]. Silica (silicon dioxide, SiO_2_) accumulates abundantly in plant cells in the form of amorphous silica in the cell walls of leaves and stems, with some being deposited in the inner cortex cells of roots [5,6], forming a physical barrier. It not only enhances tissue strength [7] but also alleviates biotic (pathogen infection and pest attack) and abiotic (salinity, high temperature, drought, heavy metals, nutrient imbalance, and UV−B radiation) stress to plants by regulating photosynthesis, nutrient uptake, and levels of antioxidants, hormones, and gene expression, thus improving crop yield and quality [8,9,10,11,12,13,14,15,16,17]. In studies on the relation of Si and photosynthesis, Si uptake by plants was found to alleviate stress−induced photosynthetic inhibition to varying degrees. Si enhances photosynthesis by balancing nutrients, regulating gas exchange, altering the levels of phytohormones, increasing leaf water content, enhancing antioxidant content, and controlling gene expression [12,18,19]. In recent years, over−cultivation has led to increasing deficiencies in essential elements. In particular, growth restriction and diseases caused by Ca deficiency are important limiting factors in tomato production, prompting active research for ways to mitigate them. The unique role of Si in mitigating nutrient imbalances has attracted increasing attention [20]. Some studies have shown that supplementation with Si can provide some relief in case of excess or deficiency of certain essential elements, and hence, several countries have begun to pay attention to the role of Si in practical production [21]. Si might have the potential to alleviate mild Ca deficiency by improving root growth and the efficiency of Ca utilization under Ca deficient conditions [15,22], thus improving tomato yield and quality [23].

Of note, Ca is essential for the growth and development of plants [24]. It is involved in various pathways of plant growth and metabolism, such as the biosynthesis of cell walls and organelles, photosynthesis [25], adversity stress [26], protein regulation [27], hormone response [28], uptake and transport of ions [29], and other physiological and biochemical responses, as well as the establishment of Ca signal transduction networks [30]. As an indispensable element in plant photosynthesis, Ca is also one of the most demanded elements in tomato plant cultivation [31]. Interestingly, Ca plays an important role in various processes of the photosynthetic pathway, such as in the mechanism of stomatal movement [32] as a cofactor involved in the formation of photosystem II (PSII) activation sites, in the regulation of the activity of photosynthetic enzymes for carbon assimilation [33,34], and also in the regulation of photosynthetic protection [35]. Photosynthesis, which is the basis of plant growth, has a high sensitivity to stress [36]. Consequently, Ca deficiency leads to reduced photosynthetic capacity, ultimately causing a decline in crop yield and quality. Therefore, the detrimental effect of Ca deficiency on the photosynthetic capacity of plants cannot be ignored. 

China is largely an agricultural country with the largest tomato cultivation area and market share worldwide [37,38]. However, the continuous development of agriculture has given rise to problems such as soil nutrient loss, among which Ca deficiency in tomato plants is particularly common and generally unpreventable by Ca application [39]. Ca deficiency in tomato plants is generally manifested by the yellowing and wilting of leaf margins of functional leaves and the gradual necrosis, curling, and deformation of meristematic tissues such as terminal buds, lateral buds, and root tips [40], leading to plant physiological diseases such as reduced photosynthetic capacity [41] and oxidative stress [42]. In addition, Ca deficiency also causes a decrease in pectin Ca, leading to tissue degeneration and deformation, seriously affecting fruit appearance and intrinsic quality. Therefore, alleviating Ca deficiency is important for tomato growth, fruit quality, and transport storage [43]. At present, Si fertilizers have been mainly tested on various types of grass, and most of these studies have focused on the effects of Si on the antioxidant system. There have been relatively few studies on the Si−induced mitigation of the photosynthetic inhibition under low−Ca stress in tomato plants, and hence, the interaction between Si and Ca in plant growth needs further in−depth study.

The protective effect of Si on plant photosynthesis reported in studies exploring the Si−induced mitigation of plant stresses, both biotic and abiotic, has received increasing attention. However, studies on the role of different Si application methods on the photosynthetic capacity, related gene expression, and regulation of the activities of related enzymes during carbon assimilation in tomato plants under low−Ca stress have been lacking. Therefore, this study was conducted to further elucidate the mechanisms of action of different Si application methods on the photosynthetic system of the tomato plant under low−Ca stress by assessing their effects on the growth status, leaf photosynthetic capacity, adenosine triphosphate (ATP) content, stomatal state, Calvin cycle−related enzymatic activity, and gene expression in tomato seedlings under low−Ca stress.

## 2. Results

### 2.1. Effect of Exogenous Si on Growth Parameters in Tomato under Low−Ca Stress

**Figure 1:**
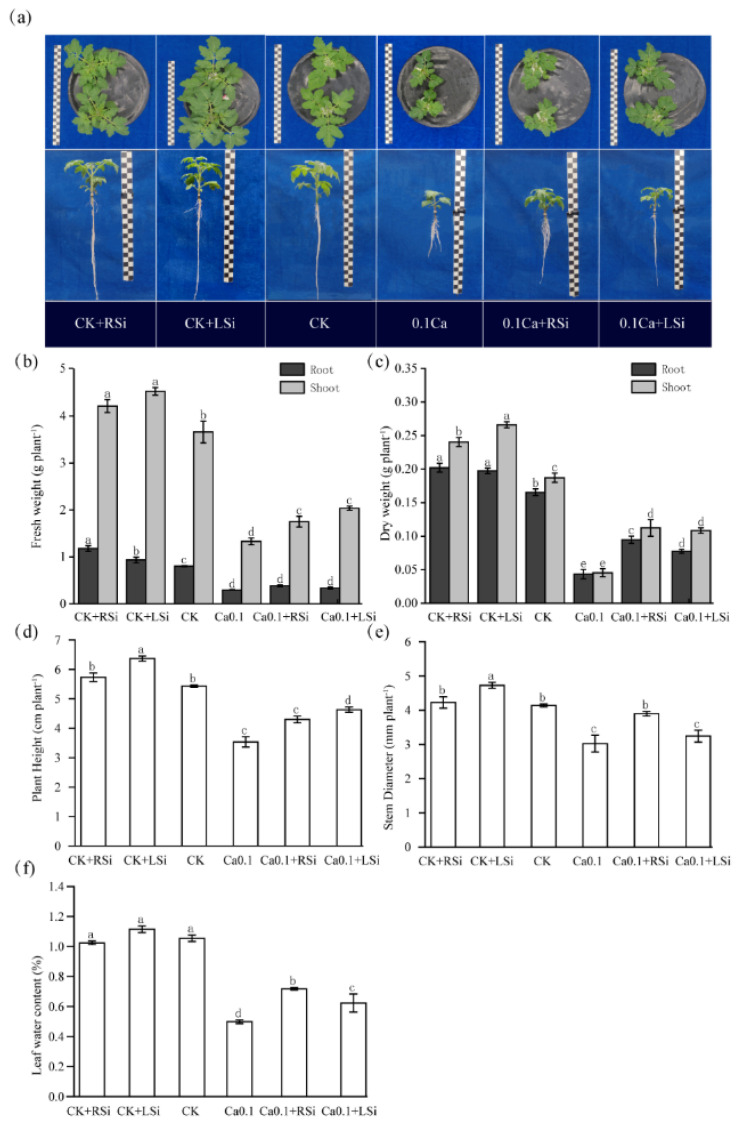
Effect of exogenous Si on growth parameters in tomato under low−calcium stress. (**a**) Plant morphologies of tomato seedlings after different treatments; (**b**) plant height; (**c**) stem diameter; (**d**) fresh weight; (**e**) dry weight; (**f**) leaf water content. The means of three replicates ± standard error is shown. The data were subjected to an ANOVA test to determine the LSD and bars superscribed by different letters are significantly different at *p* < 0.05. Standard errors are indicated by bars. CK + RSi: 4 mM Ca in Hoagland nutrient solution + root application of 1 mM Si; CK + LSi: 4 mM Ca in Hoagland nutrient solution + foliar application of 1 mM Si; CK: 4 mM Ca in Hoagland nutrient solution; 0.1Ca: 0.1 mM Ca in Hoagland nutrient solution; 0.1Ca + RSi: 0.1 mM Ca in Hoagland nutrient solution + root application of 1 mM Si; 0.1 Ca + LSi: 0.1 mM Ca in Hoagland nutrient solution + foliar application of 1 mM Si.

We found that Si application promoted growth compared with the control (CK: the nutrient solution Ca^2+^ concentration was 4 mM). The growth of tomato seedlings under low−Ca stress was significantly (*p* < 0.05) weaker; however, the addition of Si provided a certain alleviation of the growth restriction under low−Ca stress (Figure 1a). The dry and fresh weights of the tomato seedlings increased after Si application under CK treatment. The dry and fresh weights of the above—and underground shoots were significantly (*p* < 0.05) increased by Si spraying (CK + LSi), while the fresh weight of the roots was significantly (*p* < 0.05) increased by Si application (CK + RSi). The dry and fresh weight of the above− and underground tomato plant biomass under low calcium (0.1Ca) treatment was significantly (*p* < 0.05) decreased compared with CK, and Si application significantly alleviated the depressed growth under 0.1Ca treatment. However, there were no significant differences between different Si application methods. Si application under adequate calcium conditions during CK treatment was beneficial to the growth of tomato seedlings, but the increase in dry weight was not as significant as those of the 0.1Ca + RSi and 0.1Ca + LSi treatments (Figure 1b,c). 

We found that compared with CK, the plant height and stem diameter of tomato seedlings under CK + LSi treatment were significantly (*p* < 0.05) increased, while the plant height, stem diameter, and leaf water content of tomatoes under 0.1Ca treatment were significantly (*p* < 0.05) decreased by 35%, 21%, and 57%, respectively. Compared with the 0.1Ca treatment, the plant height of tomatoes under 0.1Ca + LSi significantly (*p* < 0.05) increased by 31% (Figure 1d). We also observed that stem diameter was significantly (*p* < 0.05) increased by 29% upon the 0.1Ca + RSi treatment compared with that upon the 0.1Ca treatment (Figure 1e). We further observed that low−Ca stress significantly (*p* < 0.05) reduced leaf water content, with the 0.1Ca treatment resulting in leaf water content less than half of that in CK, whereas 0.1Ca + RSi and 0.1Ca + LSi significantly (*p* < 0.05) increased leaf water content by 44% and 25% compared with that in plants after 0.1Ca treatment (Figure 1f).

### 2.2. Effect of Exogenous Si on Tomato Root System under Low−Ca Stress

**Figure 2:**
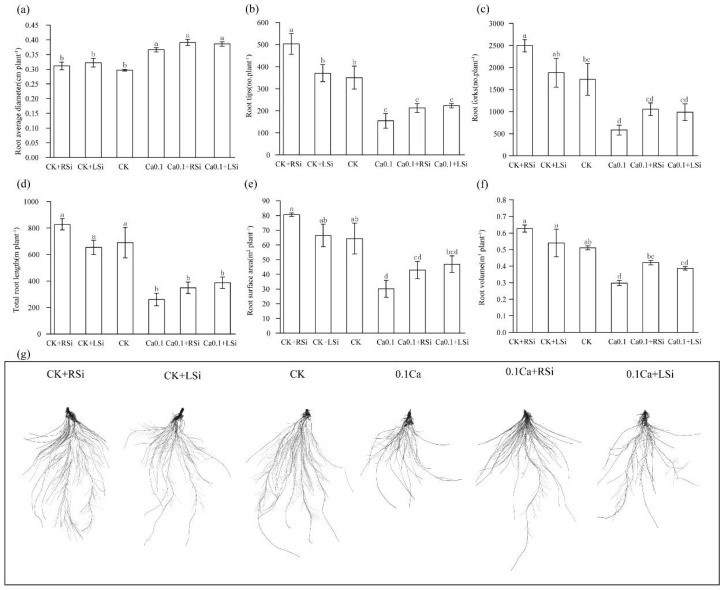
Effect of exogenous Si on tomato root system under low−calcium stress. (**a**) Root average diameter; (**b**) root tips; (**c**) root forks; (**d**) total root length; (**e**) root surface area; (**f**) root volume; (**g**) scan image of root morphology. The means of three replicates ± standard error is shown. The data were subjected to an ANOVA test to determine the LSD, and bars superscribed by different letters are significantly different at *p* < 0.05. Standard errors are indicated by bars. CK + RSi: 4 mM Ca in Hoagland nutrient solution + root application of 1 mM Si; CK + LSi: 4 mM Ca in Hoagland nutrient solution + foliar application of 1 mM Si; CK: 4 mM Ca in Hoagland nutrient solution; 0.1Ca: 0.1 mM Ca in Hoagland nutrient solution; 0.1Ca + RSi: 0.1 mM Ca in Hoagland nutrient solution + root application of 1 mM Si; 0.1Ca + LSi: 0.1 mM Ca in Hoagland nutrient solution + foliar application of 1 mM Si.

We found that compared to CK, CK + RSi treatment significantly (*p* < 0.05) increased the number of root tips and root fractions of the tomato seedlings, but the 0.1Ca treatment significantly (*p* < 0.05) reduced the total root length, root tip number, root surface area, and the number of root forks by 62%, 56%, 53%, and 66%, respectively (Figure 2). The mean root diameters in plants after 0.1Ca, 0.1Ca + RSi, and 0.1Ca + Lsi treatments were significantly (*p* < 0.05) greater than those in CK, increasing by 23%, 32%, and 30%, respectively (Figure 2d). In addition, compared with CK, root volumes were reduced by 42% and 24% upon 0.1Ca and 0.1Ca + LSi treatments, respectively. We detected that 0.1Ca + RSi treatment significantly (*p* < 0.05) ameliorated the reduction in root volume of tomato plants under low−Ca stress (Figure 2c). Imaging of the scanned roots revealed that the number of root fractions and root tips under the CK, CK + RSi, and CK + LSi treatments were significantly higher than those of other treatments, and this increase was more prominent under the CK + RSi treatment. In contrast, the number of main root branches was significantly (*p* < 0.05) reduced after 0.1Ca treatment, explaining the lower average diameter of roots in CK. We observed that the differences in root length and the number of root forks between the 0.1Ca treatment and CK groups were significant, with Si application significantly promoting root growth and development (Figure 2g).

### 2.3. Effect of Exogenous Si on Chlorophyll Content of Tomato Leaves under Low−Ca Stress

**Figure 3:**
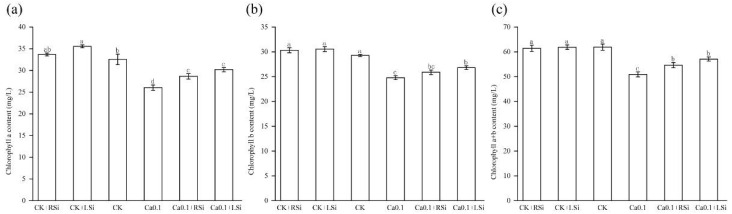
Effect of exogenous Si on chlorophyll content of tomato leaves under low−calcium stress. (**a**) Chlorophyll a content; (**b**) chlorophyll b content; (**c**) chlorophyll a + b content. The means of three replicates ± standard error is shown. The data were subjected to an ANOVA test to determine the LSD and bars superscribed by different letters are significantly different at *p* < 0.05. Standard errors are indicated by bars. CK + RSi: 4 mM Ca in Hoagland nutrient solution + root application of 1 mM Si; CK + Lsi: 4 mM Ca in Hoagland nutrient solution + foliar application of 1 mM Si; CK: 4 mM Ca in Hoagland nutrient solution; 0.1Ca: 0.1 mM Ca in Hoagland nutrient solution; 0.1Ca + Rsi: 0.1 mM Ca in Hoagland nutrient solution + root application of 1 mM Si; 0.1Ca + Lsi: 0.1 mM Ca in Hoagland nutrient solution + foliar application of 1 mM Si.

We found that the chlorophyll a, b, and a + b contents of tomato leaves treated with CK, CK + RSi, and CK + LSi treatments showed little difference, but those of tomato leaves under 0.1Ca treatment were significantly (*p* < 0.05) reduced compared with those in CK by 20%, 15%, and 18%, respectively. Conversely, compared with 0.1Ca, the chlorophyll a and b contents in tomato leaves treated with 0.1Ca + LSi were significantly (*p* < 0.05) increased by 16% and 8%, respectively. However, we noticed that although Si application significantly (*p* < 0.05) alleviated the decrease in chlorophyll a + b content under stress and promoted the chlorophyll a content to return to the level of CK (Figure 3a,b), the difference in the alleviation effect between the two types of Si application was not significant (Figure 3c).

### 2.4. Effect of Exogenous Si on Stomatal Morphology and Photosynthetic Characteristics of Tomato Leaves under Low−Ca Stress

**Figure 4:**
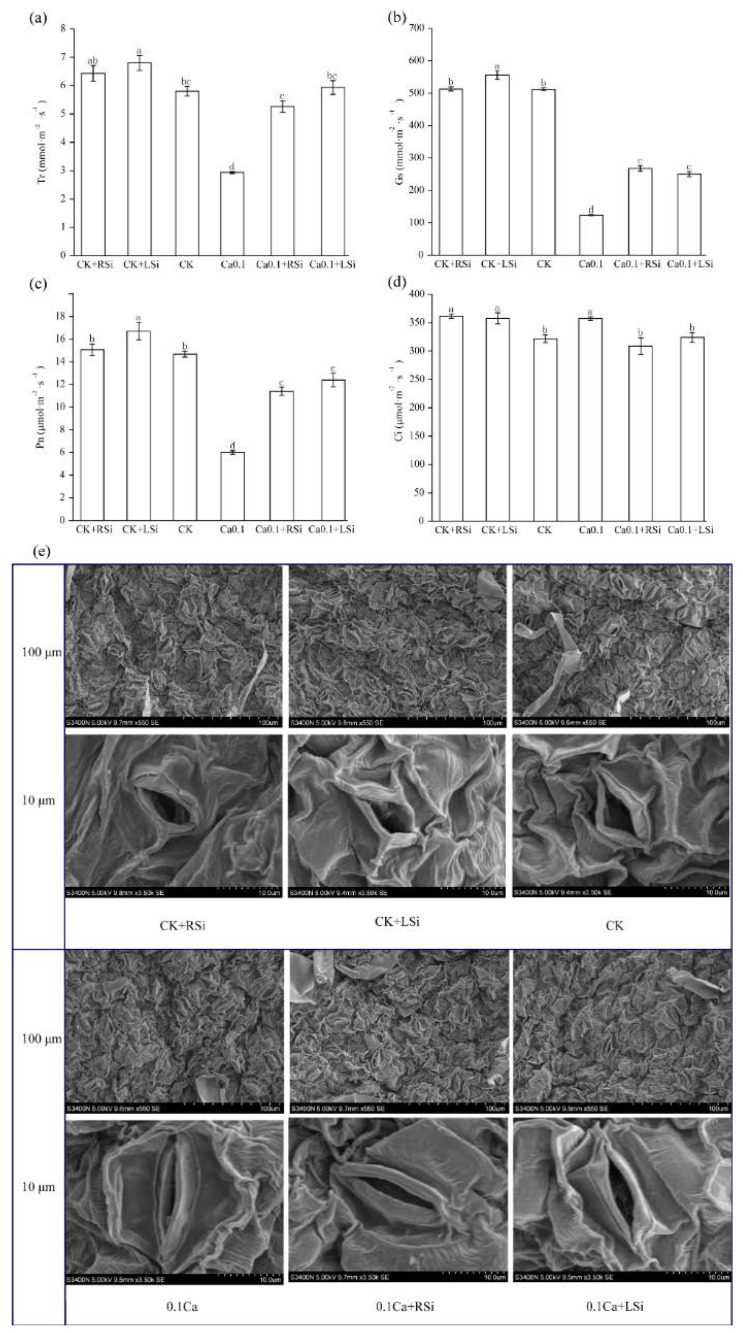
Effect of exogenous Si on stomatal morphology and photosynthetic characteristics of tomato leaves under low−calcium stress. (**a**) Transpiration rate; (**b**) stomatal conductance; (**c**) net photosynthetic rate; (**d**) intercellular carbon dioxide, (**e**) scanning electron microscope imaging of tomato leaf stomata. The means of three replicates ± standard error is shown. The data were subjected to an ANOVA test to determine the LSD, and bars superscribed by different letters are significantly different at *p* < 0.05. Standard errors are indicated by bars. CK + RSi: 4 mM Ca in Hoagland nutrient solution + root application of 1 mM Si; CK + LSi: 4 mM Ca in Hoagland nutrient solution + foliar application of 1 mM Si; CK: 4 mM Ca in Hoagland nutrient solution; 0.1Ca: 0.1 mM Ca in Hoagland nutrient solution; 0.1Ca + RSi: 0.1 mM Ca in Hoagland nutrient solution + root application of 1 mM Si; 0.1Ca + LSi: 0.1 mM Ca in Hoagland nutrient solution + foliar application of 1 mM Si.

We found that compared with CK, CK + LSi treatment significantly (*p* < 0.05) increased the transpiration rate (Tr), intercellular carbon dioxide (Ci), stomatal conductance (Gs), and net photosynthetic rate (Pn) of tomato leaves, but the Tr, Gs and Pn of tomato leaves treated with 0.1Ca were significantly (*p* < 0.05) reduced by 49%, 50%, and 59%, respectively, whereas, Si application significantly (*p* < 0.05) increased the Tr under low−Ca stress, causing it to approach the levels in CK (Figure 4a). Compared with 0.1Ca, Gs was significantly (*p* < 0.05) increased by 116% and 100% after 0.1Ca + RSi and 0.1Ca + LSi treatments, respectively (Figure 4b). However, compared with CK, Pn was decreased by 57%, 21%, and 14% after 0.1Ca, 0.1Ca + RSi, and 0.1Ca + LSi treatments, respectively (Figure 4c). We observed that although Si application significantly (*p* < 0.05) increased the Gs and Pn under low−Ca stress compared with those in CK, the increases due to different Si application methods were not significantly different. We noticed that 0.1Ca treatment led to the highest (Ci) content, which was significantly (*p* < 0.05) higher than that after other treatments, with an increase of 11% compared with that in CK (Figure 4c). Si application also increased the Pn, Gs, and Tr, thereby reducing the Ci. We observed the stomatal morphology of plants under different treatments using scanning electron microscopy and found that the stomata opening of tomato leaves under CK, CK + RSi, and CK + LSi treatments were similar. However, under low calcium stress, the stomata closed to different degrees, and the number of stomata was relatively low. Conversely, we found that Si application effectively alleviated stomatal closure, with the number of stomata and stomatal openings being significantly (*p* < 0.05) greater than those in non−Si−treated plants (Figure 4e).

### 2.5. Effect of Exogenous Si on Fluorescence Parameters of Tomato Leaves under Low−Ca Stress

**Figure 5:**
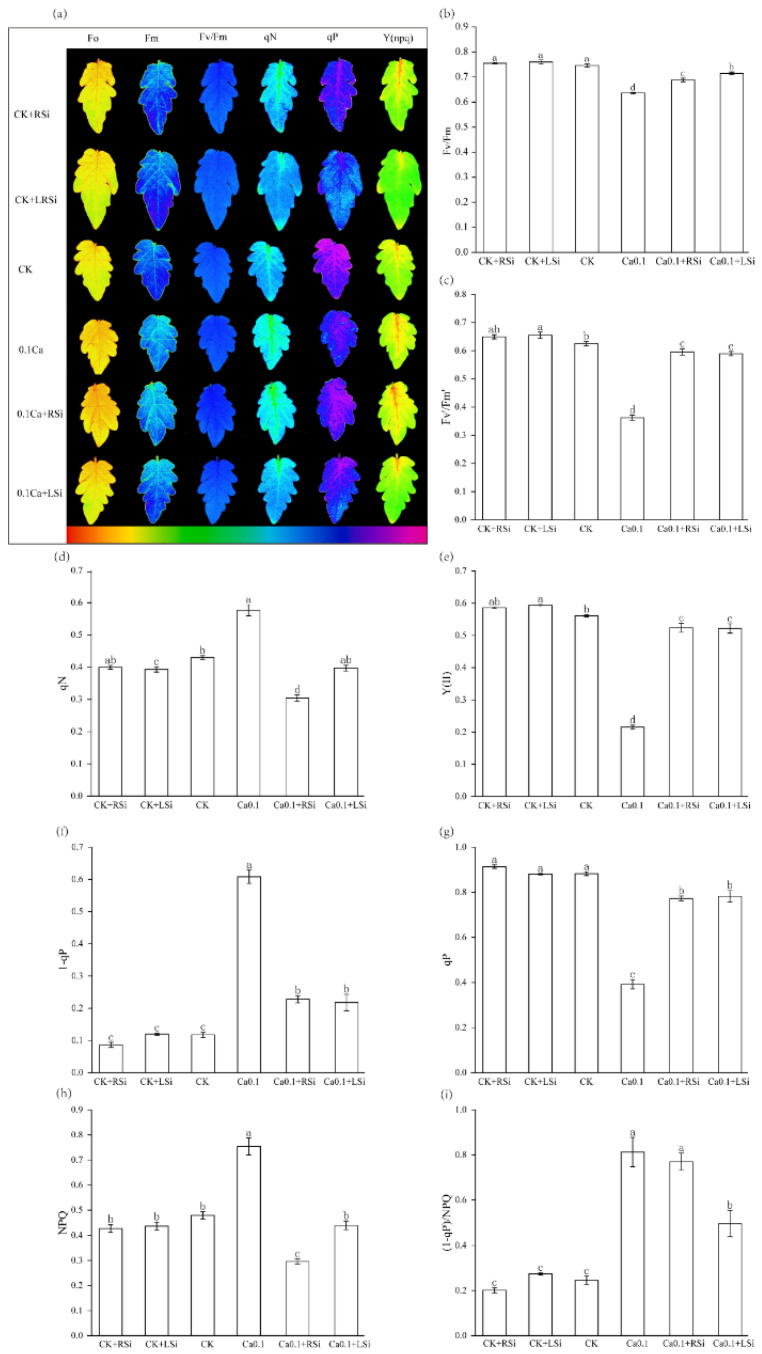
Effect of exogenous Si on fluorescence parameters of tomato leaves under low−calcium stress. (**a**) Fluorescence imaging of tomato leaf; (**b**) Fv/Fm: maximum photochemical efficiency; (**c**) Fv′/Fm′: effective photochemical quantum yield; (**d**) qN: nonphotochemical quenching coefficient; (**e**) Y(II): actual photochemical efficiency; (**f**) 1 − qP: PSII excitation pressure; (**g**) qP: photochemical quenching coefficient, (**h**) NPQ: nonphotochemical quenching coefficient; (**i**) (1 − qP)/qN: excess excitation energy. The means of three replicates ± standard error is shown. The data were subjected to an ANOVA test to determine the LSD, and bars superscribed by different letters are significantly different at *p* < 0.05. Standard errors are indicated by bars. CK + RSi: 4 mM Ca in Hoagland nutrient solution + root application of 1 mM Si; CK + LSi: 4 mM Ca in Hoagland nutrient solution + foliar application of 1 mM Si; CK: 4 mM Ca in Hoagland nutrient solution; 0.1Ca: 0.1 mM Ca in Hoagland nutrient solution; 0.1Ca + RSi: 0.1 mM Ca in Hoagland nutrient solution + root application of 1 mM Si; 0.1Ca + LSi: 0.1 mM Ca in Hoagland nutrient solution + foliar application of 1 mM Si.

We found that the Fv′/Fm′ and Y(II) of tomato leaves were significantly (*p* < 0.05) increased, but the qN of tomato leaves was significantly (*p* < 0.05) decreased after Si spraying under adequate calcium conditions. However, the degree of change was not as significant as that under low calcium conditions. The maximum photochemical efficiency (Fv/Fm), effective photochemical quantum yield (Fv′/Fm′), photochemical quenching coefficient (qP), and actual photochemical efficiency [Y(II)] of tomato leaves treated with 0.1Ca were significantly (*p* < 0.05) lower by 15%, 42 %, 56%, and 62%, respectively, compared with those in CK. Conversely, Si application effectively alleviated the decreases in the above parameters. In particular, we found that 0.1Ca + LSi treatment was significantly (*p* < 0.05) more effective than 0.1Ca + RSi treatment in alleviating Fv/Fm under low−Ca stress (Figure 5b,c,e,g). In addition, the nonphotochemical quenching coefficient (qN), the quantum yield of nonphotochemical quenching [Y(NPQ)], and PSII excitation pressure (1 − qP) values were significantly (*p* < 0.05) higher under low−Ca stress compared with those in CK, except for excess excitation energy [(1 − qP)/NPQ]; whereas, Si application significantly (*p* < 0.05) reduced the increasing trend of these parameters, with Si spraying being more effective (Figure 5d,f,h,i). The color differences of all parameters in images were consistent with the trends of the corresponding parameters (Figure 5a).

### 2.6. Effect of Exogenous Si on ATPase Activity and ATP Content of Tomato Chloroplasts under Low−Ca Stress

**Figure 6:**
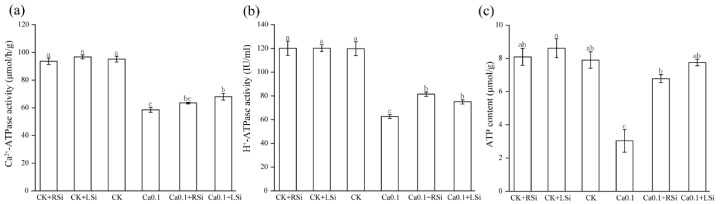
Effect of exogenous Si on chloroplast ATPase activity and ATP content in tomato under low−calcium stress. (**a**) Ca^2+^−ATPase activity; (**b**) H+−ATPase activity; (**c**) ATP content. The means of three replicates ± standard error is shown. The data were subjected to an ANOVA test to determine the LSD, and bars superscribed by different letters are significantly different at *p* < 0.05. Standard errors are indicated by bars. CK + RSi: 4 mM Ca in Hoagland nutrient solution + root application of 1 mM Si; CK + LSi: 4 mM Ca in Hoagland nutrient solution + foliar application of 1 mM Si; CK: 4 mM Ca in Hoagland nutrient solution; 0.1Ca: 0.1 mM Ca in Hoagland nutrient solution; 0.1Ca + RSi: 0.1 mM Ca in Hoagland nutrient solution + root application of 1 mM Si; 0.1Ca + LSi: 0.1 mM Ca in Hoagland nutrient solution + foliar application of 1 mM Si.

The activities of Ca^2+^−ATPase, H^+^−ATPase, and the ATP content in tomato leaves treated with CK + RSi and CK + LSi were not significantly different from those of the CK group. However, the H^+^−ATPase activity was significantly (*p* < 0.05) reduced by 48%, 32%, and 37%, and the Ca^2+^−ATPase activity was significantly (*p* < 0.05) reduced by 38%, 22%, and 28% after 0.1Ca, 0.1Ca + RSi, and 0.1Ca + LSi treatments, respectively, compared with those in CK. Low−Ca stress significantly (*p* < 0.05) reduced the activities of H^+^−ATPase and Ca^2+^−ATPase of tomato leaves, whereas Si application effectively alleviated these reductions; however, the alleviation effect of different Si application methods did not differ significantly (Figure 6a,b). Likewise, we noticed that low−Ca stress significantly (*p* < 0.05) reduced the ATP content, dropping to only 52% of that in CK after 0.1Ca treatment but was restored to the levels shown in CK at normal Ca levels after Si application (Figure 6c).

### 2.7. Effect of Exogenous Si on Photosynthesis−Related Enzymatic Activities in Tomato under Low−Ca Stress

**Figure 7:**
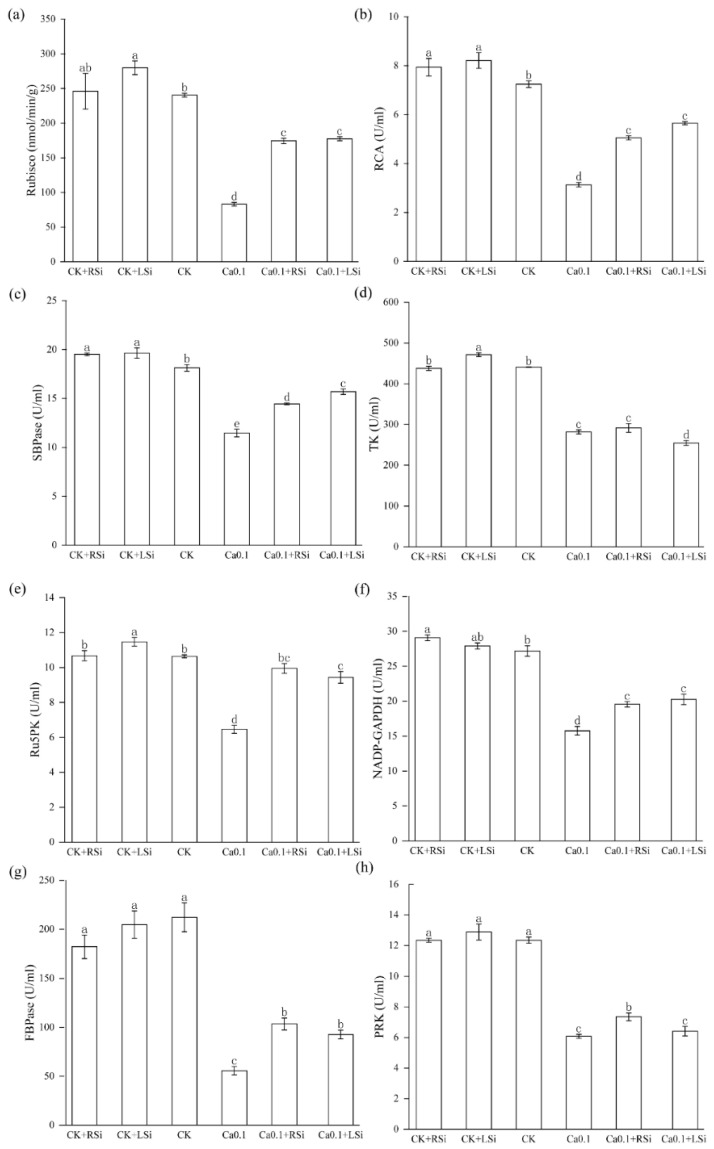
Effect of exogenous Si on the activity of enzymes related to photosynthesis in tomato plants under low−calcium stress. (**a**) RuBisco, (**b**) RCA, (**c**) SBPase, (**d**) TK, (**e**) Ru5PK, (**f**) NADP−GAPDH, (**g**) FBPase, (**h**) PRK. The means of three replicates ± standard error is shown. The data were subjected to an ANOVA test to determine the LSD, and bars superscribed by different letters are significantly different at *p* < 0.05. Standard errors are indicated by bars. CK + RSi: 4 mM Ca in Hoagland nutrient solution + root application of 1 mM Si; CK + LSi: 4 mM Ca in Hoagland nutrient solution + foliar application of 1 mM Si; CK: 4 mM Ca in Hoagland nutrient solution; 0.1Ca: 0.1 mM Ca in Hoagland nutrient solution; 0.1Ca + RSi: 0.1 mM Ca in Hoagland nutrient solution + root application of 1 mM Si; 0.1Ca + LSi: 0.1 mM Ca in Hoagland nutrient solution + foliar application of 1 mM Si.

We found that compared with CK, the activities of RCA and SBPcase in tomato leaves treated with CK + RSi and CK + LSi were significantly (*p* < 0.05) increased. The activities of Rubisco, TK, and Ru5PK in tomato leaves treated with CK + LSi were also significantly (*p* < 0.05) increased. However, the activities of Rubisco, RCA, SBPcase, TK, Ru5PK, NADP−GAPDH, FBPase, and PRK were significantly (*p* < 0.05) reduced in tomato leaves treated with 0.1Ca compared with those in CK (Figure 7). Likewise, the activities of all these enzymes except TK and PRK were significantly (*p* < 0.05) increased after Si application compared with those after 0.1Ca treatment. We also found that the activities of SBPase after 0.1Ca + LSi treatment were significantly (*p* < 0.05) higher than those after 0.1Ca + RSi treatment, whereas those of TK and PRK were increased after root application of Si. These results indicate that low−Ca stress severely affected photosynthesis−related enzymatic activities, which were effectively alleviated after applying exogenous Si in plants under low−Ca stress. Moreover, we found that Si promoted photosynthetic carbon assimilation and alleviated growth inhibition in tomato plants under low−Ca stress by increasing the activities of Calvin cycle−related enzymes.

### 2.8. Effect of Exogenous Si on the Expression of Genes Related to Photosynthetic Enzymatic Activity in Tomato under Low−Ca Stress

**Figure 8:**
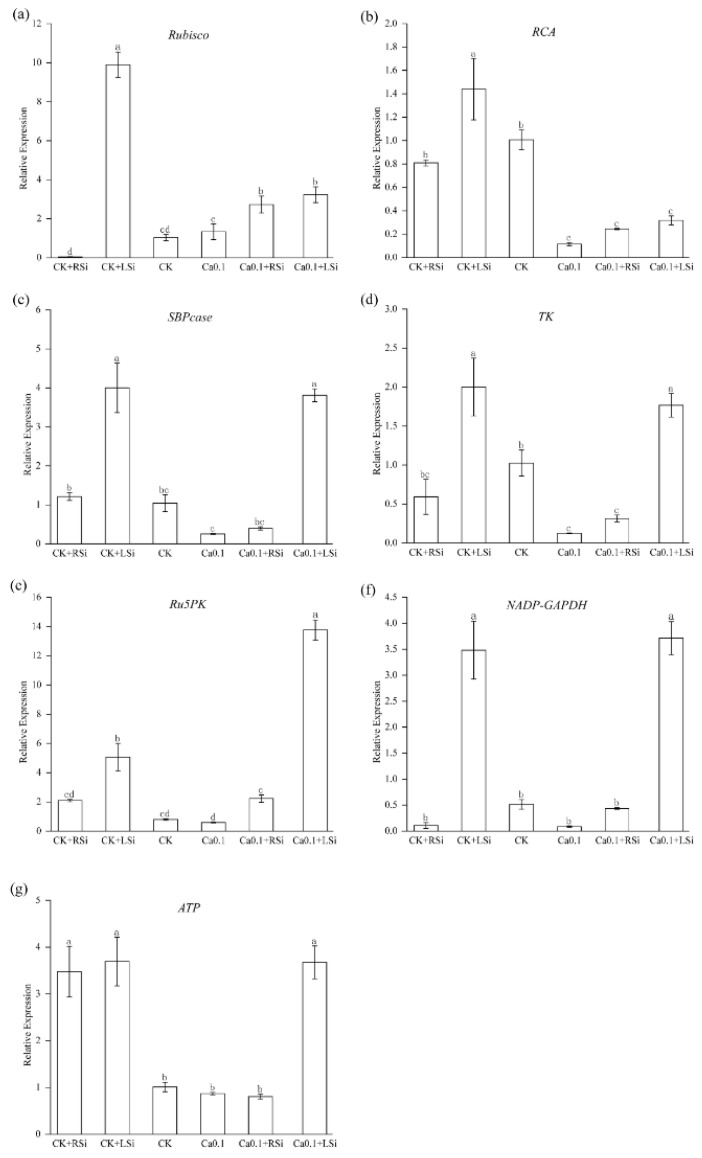
Effect of exogenous Si on the expression of photosynthetic related genes in tomato under low calcium stress. (**a**) *Rubisco*: rubisco coenzyme; (**b**) *RCA*: ribulose diphosphate carboxylase/oxygenase activating enzyme or rubisco activating enzyme; (**c**) *SBPcase*: 1,7−diphosphoglycosylase; (**d**) *TK*: transketolase; (**e**) *Ru5PK*: ribulose 5−phosphate kinase; (**f**) *NADP−GAPDH*: 3−phosphoglycerate dehydrogenase; (**g**) *ATP*: ATP synthase subunit. The means of three replicates ± standard error is shown. The data were subjected to an ANOVA test to determine the LSD, and bars superscribed by different letters are significantly different at *p* < 0.05. Standard errors are indicated by bars. CK + RSi: 4 mM Ca in Hoagland nutrient solution + root application of 1 mM Si; CK + LSi: 4 mM Ca in Hoagland nutrient solution + foliar application of 1 mM Si; CK: 4 mM Ca in Hoagland nutrient solution; 0.1Ca: 0.1 mM Ca in Hoagland nutrient solution; 0.1Ca + RSi: 0.1 mM Ca in Hoagland nutrient solution + root application of 1 mM Si; 0.1Ca + LSi: 0.1 mM Ca in Hoagland nutrient solution + foliar application of 1 mM Si.

We found that compared with CK, CK + LSi treatment significantly increased the expression of related *Rubisco*, *RCA*, *SBPcase*, *TK*, *Ru5PK*, and *NADP−GAPDH* enzymes genes, while both CK + RSi and CK + LSi treatments increased the expression of *ATP*−related genes in tomato seedling leaves. But the levels of expression of *SBPcase*, *RCA*, *TK*, and *NADP−GAPDH* genes, which are involved in regulating photosynthesis−related enzymatic activities, were significantly (*p* < 0.05) reduced under low−Ca stress, with the gene expression of RCA showing the greatest decrease, as indicated by qRT−PCR. Conversely, the root application of Si significantly (*p* < 0.05) upregulated the expression of *Rubisco* and *Ru5PK* compared with that in CK (Figure 8a,e). Similarly, foliar spraying of Si significantly (*p* < 0.05) upregulated the gene expression levels of *Rubisco*, *RCA*, *SBPcase*, *TK*, *Ru5PK*, *NADP−GAPDH*, and *ATP*. We concluded that the increased expression of genes related to the regulation of the activity of key photosynthetic enzymes after Si treatment might be one of the main reasons for enhanced photosynthesis.

## 3. Discussion

Ca is involved in many processes of plant growth, including the effective maintenance of plant cell structure [44]. As Ca is not free−flowing in plants, short periods of Ca deficiency can rapidly affect actively growing tissues, resulting in slowed growth [45,46]. Si has been shown to exert extraordinary effects on plant growth and development [47,48] and can also alleviate deficiencies of other elements by altering the uptake and accumulation of minerals [49,50]. In our study, the plant height, stem diameter, dry and fresh weight, leaf water content, and root growth were significantly reduced under low−Ca stress (Figure 1 and Figure 2), in consistency with the results of previous studies. When Si was administered to tomato seedlings under adequate calcium concentrations, the plant height, stem diameter, dry weight, and leaf water content increased. However, these effects were more significant under low calcium stress, and Si appeared to alleviate low calcium−induced reduced growth. These results indicate that Si has a role in promoting mineral absorption. More specifically, in our study, root Si application in plants under low−Ca stress significantly increased stem diameter, increased dry weight and promoted root growth. In contrast, foliar spraying of Si significantly increased above−ground dry weight, similar to the Si−induced root growth promotion in studies on sorghum [51]. Si application might alleviate the inhibition of plant growth under abiotic stress by alleviating the induced oxidative stress and regulating nutrient uptake, consistent with previous studies [15,52,53].

Chlorophyll is essential for converting light energy to chemical energy in plants, and its reduction limits the photosynthetic process [54]. In addition, the opening and closing of stomata, which serve as inlets for CO_2_, the raw material for photosynthesis, is essential for facilitating the exchange of gases between the above−ground parts of the plant and the atmosphere [55]. Therefore, photosynthetic characteristics such as Ci, Tr, Gs, and Pn are closely related to stomatal opening and closing. Stomatal movement has been associated with Ca^2+^ [56], which has also been found to alleviate the low oxygen−induced reductions in Pn, Gs, and Ci in cucumbers [57]. In our study, we found that Ca deficiency not only affected leaf stomatal movement but also inhibited photosynthetic properties and chlorophyll biosynthesis. Likewise, low−Ca stress significantly reduced chlorophyll content, stomatal opening, Tr, Gs, and Pn, whereas it significantly increased Ci in tomato plants. Under adequate calcium conditions, only Tr, Gs, and Pn were significantly increased by foliar Si spraying, while root Si application did not significantly affect the Tr, Gs, Pn, or chlorophyll contents of tomato seedlings (Figure 3 and Figure 4). The application of appropriate levels of Si is known to increase the chlorophyll content and stomatal conductance in tomato plants [51,58]. In this study, we also found that foliar Si spraying had better positive effects on the Tr, Gs, and Pn of tomato leaves under adequate calcium conditions. Foliar Si spraying promoted the growth of tomato leaves. Under low calcium conditions, there were no significant differences between the two Si application methods, which may be because both Si application methods can alleviate stress by promoting calcium absorption. The chlorophyll content of tomato leaves was also significantly increased by Si application, but only under low calcium conditions.

Chlorophyll fluorescence parameters respond to the absorption, conversion, transfer, and distribution of light energy by plants and can be used to monitor the effects of environmental factors on plant photosynthesis. Fluorescence parameters, such as Y(II), Fv/Fm, Fv′/Fm′, qP, Y(NPQ), NPQ, 1 − qP/qN, and 1 − qP can accurately reflect the intensity of photosynthesis [35]. The Ca^2+^ signaling pathway has been suggested to regulate the lutein cycle−dependent NPQ [59]. Ca^2+^ is also involved in forming activation sites as PSII−related oxidoreductase cofactors [60], while the activity of Ca channels also regulates systematic changes in NPQ and 1 − qP [35]. In our study, we also found that the levels of 1 − qP/qN, 1 − qP, NPQ, and qN were significantly higher in tomato leaves after Ca deficiency (Figure 5). In contrast, both root and leaf application of Si significantly reduced them, consistent with the theory that Si reduces the dissipation of such excess light energy [58]. Si protects the photosynthetic machinery and its functions by regulating photosynthesis−related processes, such as Fv/Fm, photosystem II yield (φPSII), electron transport rate (ETR), and qP, to alleviate the stress−induced photosynthetic inhibition. We observed that the Y(II), Fv/Fm, and Fv′/Fm′ were significantly reduced due to low−Ca stress in our study. However, Si application reversed these effects and increased the chlorophyll content, photosynthesis rate, photochemical basic quantum yield (Fv/Fo), and Fv/Fm [61,62]. In our study, we found that when using the same method of Si application, the effects of Si under low calcium conditions were better than those under adequate calcium. Si application may promote the absorption and transport of calcium and reduce the effects of low calcium stress on the light energy utilization of tomato leaves. Both methods of Si application alleviated the photosynthetic inhibition in tomato seedlings under low−Ca stress, with a better effect observed on leaf fluorescence parameters after Si spray application. Our study was consistent with previous studies on the role of Si and Ca in plant fluorescence [35,56,63].

After completion of the first stage of the primary photosynthetic reaction, light energy is converted into electrical energy, and the generated electrons pass through a series of electron transfer bodies, causing water cleavage for oxygen and NADP^+^ reduction, and forming ATP through photosynthetic phosphorylation, finally converting electrical energy into active chemical energy. H^+^−ATPase, which is indispensable for photosynthesis, is an ATP−driven proton pump that couples ATP hydrolysis with the generation of transmembrane electrochemical proton gradients [64]. Ca^2+^ enters the cytoplasm of endodermal cells through Ca−permeable channels on the cortical side of the Casparian band, requiring cytoplasmic Ca^2+^−ATPase or intracellular Ca^2+^/H^+^−antibody pumping from the cytoplasm, and by regulating the expression and activity of these transporter proteins, Ca^2+^ can be selectively transported to the xylem at a rate consistent with above−ground requirements [44]. Ca^2+^ in chloroplasts regulates the photosynthetic pathway and is the main source of energy supply for plant cells [56]. In our study, we found that the activities of Ca^2+^−ATPase and H^+^−ATPase, as well as the ATP content, were closely related to Ca2^+^. Si’s alleviation of low calcium stress may be related to the promotion of calcium absorption and conversion by Si. The activities of Ca^2+^−ATPase and H^+^−ATPase were significantly reduced in tomato plants under low−Ca stress (Figure 6), which might have been related to the effect of low−Ca stress on the primary photosynthetic reaction. Inactivation of H^+^−ATPase has been suggested to increase the NPQ of chlorophyll fluorescence [65]. We also found a decrease in the activity of H^+^−ATPase, associated with an increased value of NPQ in tomato plants under low−Ca stress (Figure 5 and Figure 6). This decrease in the Y(II) affected the formation of assimilatory forces, such as ATP and NADPH, resulting in a decrease in the photosynthetic electron transfer rate. Our simulated low−Ca environment significantly reduced the ATP content of tomato leaves, whereas Si application significantly increased the content of Ca^2+^−ATPase with H^+^−ATPase and ATP in the stress environment, contributing to a smooth Calvin cycle. In addition, some H^+^−ATPases have also been shown to play an important role in regulating root hair growth, root sheath formation, and inter−root microbiome composition [66]. These findings were confirmed in our study, in which the H^+^−ATPase content was increased, and root hair growth was also promoted in tomato seedlings under low−Ca stress after root Si treatment (Figure 2 and Figure 6).

**Figure 9:**
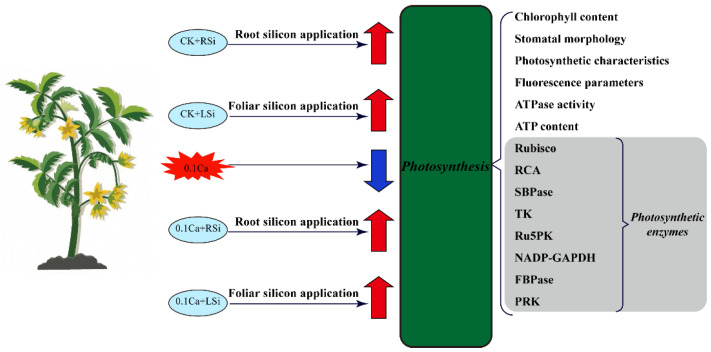
Simplified schematic diagram of photosynthetic response of tomato seedlings to 0.1Ca, 0.1Ca + RSi, and 0.1Ca + LSi. Negative regulation is indicated by the blue down arrow, Positive regulation is indicated by the red up arrow. CK + RSi: 4 mM Ca in Hoagland nutrient solution + root application of 1 mM Si; CK + LSi: 4 mM Ca in Hoagland nutrient solution + foliar application of 1 mM Si; 0.1Ca: 0.1 mM Ca in Hoagland nutrient solution; 0.1Ca + RSi: 0.1 mM Ca in Hoagland nutrient solution + root application of 1 mM Si; 0.1Ca + LSi: 0.1 mM Ca in Hoagland nutrient solution + foliar application of 1 mM Si. RuBPCase: ribulose 1,5−bisphosphate carboxyhase; RCA: rubisco activation enzyme; SBPase: 1,7−diphosphoric acid cingulum heptulosylase; TK: transketolase; Ru5PK: Ribulose 5−phosphate kinase; NADP−GAPDH, glyceraldehyde−3−phosphate dehydrogenase; FBPase: Fructose−1,6−bisphosphatase; PRK: Ribulose−5−phosphate kinase.

The Calvin cycle is central to photosynthetic carbon assimilation and occurs in the chloroplast stroma, with rubisco, FBPase, and SBPase dominating it. In particular, SBPase activity is known to be the main determinant affecting the photosynthetic capacity of seedlings. RCA is a rubisco−activating enzyme; the activity of rubisco in plants depends on its activation by RCA. TK in chloroplasts is another key enzyme of the Calvin cycle. It is involved in the regeneration of various metabolites in the Calvin cycle, phosphorylated in chloroplast extracts, and presumably related to the Ca^2+^−dependent pathway [34]. In addition, two nonregulatory enzymes, TK and aldolase, the activities of which are regulated by Ca^2+^ [67], have also been shown to potentially contribute to carbon fluxes in the Calvin cycle [68]. Ca also is known to regulate the Calvin cycle by mediating the formation of the PRK/GAPDH/CP12 complex [69]. In practice, Ca deficiency usually causes crop deficiency disorders and growth abnormalities, such as growing point necrosis and functional leaf deformities, which seriously affect photosynthesis [11]. Moderate amounts of Si have been reported to increase chlorophyll content and improve the photosynthetic rate in crops under abiotic stress [58,70,71]. Under our simulated low calcium stress, the activities of Calvin cycle−related enzymes rubisco, SBPcase, FBPcase, RCA, TK, NADP−GAPDH, Ru5PK, and PRK were significantly reduced (Figure 7). Moreover, the levels of expression of key enzymes in the Calvin cycle were also significantly reduced (Figure 9). This might be because Ca regulates the transcription and translation of genes related to photosynthesis (chloroplast proteins and enzymes) [56]. In contrast, we found that Si treatment significantly increased the activity of key enzymes in the Calvin cycle and the levels of expression of genes related to these enzymes, as well as those of ATPase−related genes (Figure 7 and Figure 9). Si application significantly promoted the activity and gene expression of many photosynthesis−related enzymes under both low calcium and adequate calcium conditions. This coincided with previous studies on the effect of Si in increasing the expression of photosynthesis−related genes [18,48,72]. Interestingly, foliar spray Si treatments were the most effective; this might be attributed to the direct action of silicon on leaves, thus resulting in increased plant photosynthesis and achievement of the mitigation effect at a faster rate.

Stomatal conductance, photosynthetic pigments and photosynthesis−related enzyme activities of leaves have been reported to be inhibited under low−Ca stress [32,33,34,35]. In our experiments, the photosynthesis of tomato seedlings treated with low−Ca was similarly significantly inhibited. Verma et al. indicated that Si application contributes to the improvement of crop leaf water content, stomatal conductance, CO_2_ assimilation, root growth and development, and also protects photosynthetic pigments and photosynthesis−related enzyme activities [73,74,75]. In the present experiment, we also observed that Si application in a calcium−sufficient treatment improved the growth, biomass and photosynthesis−related enzyme activities and gene expression of tomato seedlings. Nevertheless, Si application under low−Ca stress not only improved these indicators mentioned above, but also significantly increased leaf water content, photosynthetic pigment content, net photosynthetic rate, stomatal conductance, transpiration rate, photochemical efficiency, ATP activity and content, and especially the growth and development of the root system of tomato seedlings. These beneficial effects were probably due to the effectiveness of Si in promoting the expression of photosynthesis−related genes [18]. In addition, previous studies have indicated that Si can alleviate nutrient imbalance stress by promoting root growth and development [76,77]. Simultaneously, it has also been reported that Si application improves calcium use efficiency and has the potential to alleviate mild calcium deficiency [22,78]. We thus hypothesized that under low calcium stress, Si application might alleviate the growth inhibition caused with low calcium by reducing the stress on the photosynthetic system and promoting calcium uptake and translocation. It is worth noting that root−applied Si promotes growth and photosynthesis in tomato leaves but not as effectively as foliar−sprayed Si. It may be explained by the fact that Si applied in the roots first requires root uptake and xylem translocation to allow some of the Si to reach the above−ground, which then promotes above−ground growth [14,78,79,80]. From the above, it is possible that the root application of Si improves photosynthesis in tomato seedlings under low−Ca stress as a result of the root application promoting the growth and development of the root system, which in turn facilitates the uptake and transport of minerals.

## 4. Materials and Methods

### 4.1. Plant Material and Growing Conditions

We selected uniform and full tomato seeds (*Solanum lycopersicum* L. potted tomatoes; Xinxiang Shenniu Seed Co., Ltd., Henan, China) and disinfected them with 2.5% sodium hypochlorite. We then soaked them in warm water for 6 h and left them to germinate in a dark incubator at 28 °C (RDN−400E−4; Ningbo Southeast Instruments Co., Ltd., Zhejiang, China). Subsequently, uniformly germinated seeds were selected and sown in hole trays containing vermiculite. When both cotyledons of tomatoes were fully expanded, healthy, uniformly grown seedlings were selected and fixed in 1.5−L black plastic hydroponic boxes (2 plants per box). Seedlings were incubated for 7 d each with 1/8 and 1/4 times of Hoagland nutrient solution (the contents and specific nutrient solution element sources are shown in Table 1) in turn, uniformly grown established seedlings were then selected and subjected to test treatments. Sodium silicate (Na_2_SiO_3_·9H_2_O; AR, source leaf biology, Shanghai, China) was used as the Si source, Ca(NO_3_)_2_·4H_2_O (Shanghai Test, Sinopharm Chemical Reagent Co. Ltd., Shanghai, China) was used as the Ca source, and the missing N during Ca deficiency treatment was compensated with NH_4_NO_3_ (Shanghai Test, Sinopharm Chemical Reagent Co. Ltd., Shanghai, China). The experiment was conducted in an artificial climate chamber at 28/18 °C (day/night), 20,000 Lx light for 12 h, and 75% humidity. The nutrient solution was replaced every 3 d, and the pH was adjusted to 6.0 ± 0.2 with dilute hydrochloric acid.

### 4.2. Treatment and Experimental Design

In experiment 1, to determine the optimum Ca concentration causing stress without death in tomato seedlings, 2−leaf stage tomato seedlings were incubated sequentially in 1/8 and 1/4 Hoagland nutrient solutions for 7 d each and then treated with Ca at 0, 0.05, 0.1, 0.25, 0.5, 1, 2.5, 4, and 5 mM for 7 d. Based on the morphological characteristics of tomato seedlings, we found that 0.1 mM Ca caused moderate−Ca stress, whereas 4 mM Ca was optimal for tomato seedling growth (Table 2).

In Experiment 2, Hoagland nutrient solution supplemented with 0.1 mM was used to determine the appropriate concentration of Si for alleviating low−Ca stress in tomato seedlings. We tested seven Si concentrations (0, 0.25, 0.5, 0.75, 1, 1.5, and 2 mM) for both spraying and root application. For root application treatment, sodium silicate was dissolved in the nutrient solution each time it was renewed. For foliar spraying treatment, 1 mM sodium silicate solution (pH 7, supplemented with 0.01 % Tween–80) was evenly sprayed on the front and back of leaves until saturation at 10 pm each night; distilled water was sprayed in the same way for other treatments. Based on the morphological characteristics of tomato seedlings, 1.0 mM Si was found to be the most effective in relieving moderate−Ca stress in tomato seedlings (Table 3).

Based on the screening results of experiments 1 and 2, and with reference to the root and spray application of experiment 2, four treatments were set up in experiment 3: (1) 4 mM Ca in Hoagland nutrient solution + root application of 1 mM Si (CK + RSi); (2) 4 mM Ca in Hoagland nutrient solution + foliar application of 1 mM Si (CK + LSi); (3) 4 mM Ca in Hoagland nutrient solution (CK); (4) 0.1 mM Ca in Hoagland nutrient solution (0.1Ca); (5) 0.1 mM Ca in Hoagland nutrient solution + root application of 1 mM Si (0.1Ca + RSi) (6) 0.1 mM Ca in Hoagland nutrient solution + foliar application of 1 mM Si (0.1Ca + LSi). Plant morphological indicators were measured after 7 d of treatment.

### 4.3. Growth Indices

Three plants were randomly sampled from each treatment, photographed, and their respective growth conditions recorded. The plant height (from the base of the stem to the base of the petiole of the terminal leaf) and stem diameter (at 1 cm from the root) of seedlings were measured using a straightedge and vernier caliper, respectively. Tomato seedlings were cut from the rhizome junction, weighed for above− and below−ground fresh weight (FW), then placed in an oven at 105 °C for 15 min, dried at 75 °C to constant weight, and weighed again for above− and below−ground dry weight (DW) [81].

### 4.4. Leaf Relative Water Content

Fully developed functional leaves were selected, weighed (FW), completely immersed in a self−sealing bag filled with distilled water, and fully hydrated for 24 h. Then, after drying the excess water on the surface with absorbent paper, their post−hydration weight (TW) was obtained. Finally, the leaves were placed in an oven, dried at 70 °C to a constant weight, and weighed again (DW) [82].

Leaf relative water content was calculated using Equation (1):

(LWC) = (FW − DW)/(TW − DW) × 100% (1)

### 4.5. Root System Scanning

Tomato roots from each treatment were scanned using an EPSON expression 11000XL scanner (Win RHIZO Pro LA2400, Regent Instruments Inc., Quebec City, QC, Canada), and scanned photos were analyzed using the Win RHIZO 5.0 software (Regent Instruments Inc., Quebec City, QC, Canada) to obtain the total root length, root surface area, root volume, average root diameter, and the number of root tips and forks.

### 4.6. Chlorophyll Content

Chlorophyll content was determined by spectrophotometry after 95 % ethanol extraction. Absorbance was measured at 665, 649, and 470 nm according to Anwar’s method [12].

### 4.7. Determination of Photosynthetic Parameters

Under steady light intensity from 09:00 to 11:00 a.m., functional leaves of tomato seedlings at the same height were selected, and the gas exchange parameters of tomato leaves, such as Pn, Gs, Tr, and Ci, were measured using a Ciras−2 portable photosynthesizer (MA01913; PP System Inc., Amesbury, MA, USA).

### 4.8. Chlorophyll Fluorescence Parameters

After incubation in the dark for 30 min, functional tomato leaves of uniform length were selected, and the Fv/Fm was calculated by measuring the initial fluorescence (Fo), maximum fluorescence (Fm), steady−state fluorescence (Fs), maximum fluorescence yield (Fm′), and initial fluorescence (Fo′) in the light using an IMAPING−PAM modulated fluorometer (IMAPING−PAM, Walz, Effeltrich, Germany). Y(II) of PSII, qP [83], and Y(NPQ) were then calculated according to Equations (2)–(6) [84]:

(2) Fv/Fm=(Fm−Fo)/Fm

(3) $${\mathrm{Fv}}^{\prime }/{\mathrm{Fm}}^{\prime }=({\mathrm{Fm}}^{\prime }-{\mathrm{Fo}}^{\prime })/{\mathrm{Fm}}^{\prime }$$

(4) $$Y(\mathrm{II})=(({\mathrm{Fm}}^{\prime }-\mathrm{Fs})/{\mathrm{Fm}}^{\prime })$$

(5) $$\mathrm{qP}=({\mathrm{Fm}}^{\prime }-\mathrm{Fs})/({\mathrm{Fm}}^{\prime }-{\mathrm{Fo}}^{\prime })$$

(6) $$\mathrm{qN}=(\mathrm{Fm}-{\mathrm{Fm}}^{\prime })/(\mathrm{Fm}-{\mathrm{Fo}}^{\prime })$$

### 4.9. Leaf Stomatal Morphology Observation

Tomato leaves were cut into small slices (5 mm × 5 mm) (avoiding the main leaf veins) and placed in 10−mL centrifuge tubes, fully submerged in 4% glutaraldehyde fixative (Shanghai Test, Sinopharm Chemical Reagent Co. Ltd., Shanghai, China) for 2 h, and then rinsed with phosphate buffer (0.1 M PBS, pH 6.8) at 10 min intervals for 40 min. Subsequently, leaves were rinsed with a graded series of ethanol solutions (30%, 40%, 50%, 60%, 65%, 70%, and 75%) for 20 min each, soaked in 75% ethanol for 12 h, then dehydrated using another graded series of ethanol solutions (75%, 80%, 85%, 90%, and 95%) for 20 min each, and finally transferred thrice to 100% ethanol for 30 min each. After dehydration, the ethanol was removed from samples using a graded series of *tert*−butanol solutions (30%, 50%, 70%, 80%, 85%, 90%, 95%, 100%, 100%, 100%) for 30 min each. After drying, samples were sprayed with a layer of metal powder, and the stomata were observed and photographed using a scanning electron microscope (SEM; Hitachi−S3400N, Tokyo, Japan) [85].

### 4.10. Determination of Chloroplast ATPase Activity and Content

The ATP content of tomato leaves was determined using an ATP content determination kit (Suzhou Keming Biotechnology, Suzhou, China); H^+^−ATPase was determined using an H^+^−ATPase ELISA kit (Shanghai Preferred Biotechnology Co., Ltd., Shanghai, China) and Ca^2+^−ATPase was determined using a Ca^2+^−ATPase activity kit (Suzhou Keming Biotechnology Co., Ltd.).

### 4.11. Determination of Photosynthetic Enzymatic Activity

The activities of Calvin cycle key photosynthetic enzymes, RuBPCase (ribulose 1,5−bisphosphate carboxyhase), FBPase, SBPase, NADP−GAPDH, TK, RCA, PRK, and Ru5PK were determined using ELISA kits (Quanzhou Rexin Biotechnology Co., Quanzhou, China) according to the manufacturer’s instructions.

### 4.12. Determination of Relative Gene Expression

Total RNA was extracted from tomato leaves using an RNA extraction kit (Tiangen Biotechnology Co., Ltd., Beijing, China). cDNA was obtained by reverse transcription using the Fastking RT kit (Tiangen Biotechnology Co., Ltd.); corresponding primers were designed according to the CDS sequences of each gene in the tomato genome of Ensembl Plant using Primer 5.0 (Table 4). All PCR reactions were performed in a Light Cycle^®^ 96 Real−Time PCR System (Roche, Basel, Switzerland) using SYBR Primer Ex Taq^TM^ II (Tiangen Biotechnology Co., Ltd.). Reaction conditions were as follows: 40 cycles at 95 °C for 15 min, 95 °C for 10 s, 60 °C for 30 s, and 55 °C for 10 s. Relative gene expression was calculated using the 2^−∆∆CQ^ method [86].

### 4.13. Statistical Analysis

To avoid sampling chance errors, the number of replicates was set to ≥3 for all experiments. The obtained data were analyzed by one−way ANOVA using the SPSS software (version 22.0, SPSS Institute Inc., IBM Corp., New York, NY, USA). Graphical illustrations were created using Microsoft PowerPoint 2010, Origin Pro.9.0 (Origin Lab Institute Inc., Northampton, MA, USA), and Adobe Illustrator 2020.

## 5. Conclusions

Studies have shown that an environment of lower Ca concentration causes serious harm to plant growth, which is visually manifested as growth restriction. In particular, Ca deficiency reduces chlorophyll content, affects chlorophyll fluorescence, limits stomatal opening, reduces Calvin cycle−related enzymatic activity, and inhibits the expression of photosynthesis−related genes. Si application improved growth indexes, such as stem diameter, dry and fresh weight, increased photosynthetic pigment content, improved gas exchange conditions, reduced excess light energy dissipation, promoted photosynthetic phosphorylation for the conversion of electrical potential energy into ATP, enhanced Calvin cycle−related enzymatic activities such as those of rubisco, SBPcase, and FBPcase, and promoted root growth and the expression of photosynthesis−related genes, hence protecting the photosynthetic system and alleviating the low−Ca stress−induced inhibition of plant growth. Most importantly, the beneficial effects of Si on the activities of enzymes related to root development and leaf photosynthesis in tomato seedlings were greater under low calcium stress than under normal conditions. We speculate that Si may play a certain role in promoting the absorption and transport of calcium in plants to alleviate the effect of low calcium stress on tomato growth. We will study this further in future work to clarify the exact mechanisms underlying Si’s effects. Foliar spray application of Si significantly enhanced plant photosynthetic performance, whereas root application of Si promoted lateral root development. These results provided insights for the application of exogenous Si in the practical cultivation of plants in calcium−deficient environments.

## Figures and Tables

**Table 1:** Chemical composition of Hoagland nutrient solution under both normal and calcium−deficient conditions.

Source Element	Molarity (0.1 mM Ca)	Molarity (4 mM Ca)
NH_4_NO_3_	4.9 mM	1 mM
Ca(NO_3_)_2_·4H_2_O	0.1 mM	4 mM
KNO_3_	5 mM	5 mM
MgSO_4_·7H_2_O	2 mM	2 mM
KH_2_PO_4_	1 mM	1 mM
H_3_BO_3_	0.045 mM	0.045 mM
MnCl_4_·4H_2_O	0.01 mM	0.01 mM
ZnSO_4_·7H_2_O	0.8 μM	0.8 μM
H_2_MoO_4_	0.4 μM	0.4 μM
CuSO_4_·5H_2_O	0.3 μM	0.3 μM
FeSO_4_·7H_2_O	0.02 mM	0.02 mM
EDTA−Na_2_	0.02 mM	0.02 mM

**Table 2:** Calcium concentration screening.

Ca (mM)	Plant Height (cm)	Stem Diameter (mm)	Shoot		Root	
			Dry Weight (g)	Fresh Weight (g)	Dry Weight (g)	Fresh Weight (g)
0	3.167 ± 0.139 ^g^	2.497 ± 0.669 ^f^	0.149 ± 0.014 ^h^	1.633 ± 0.049 ^g^	0.0248 ± 0.0008 ^g^	0.4233 ± 0.02 ^f^
0.05	3.333 ± 0.053 ^g^	2.717 ± 0.074 ^ef^	0.176 ± 0.006 ^gh^	1.773 ± 0.033 ^g^	0.0278 ± 0.0005 ^f^	0.5833 ± 0.0145 ^e^
0.1	3.7667 ± 0.088 ^f^	3.057 ± 0.065 ^de^	0.195 ± 0.004 ^fg^	2.077 ± 0.026 ^f^	0.0333 ± 0.0005 ^e^	0.68 ± 0.0173 ^d^
0.25	4.233 ± 0.145 ^e^	3.4 ± 0.121 ^cd^	0.217 ± 0.003 ^f^	2.853 ± 0.041 ^e^	0.0393 ± 0.0005 ^d^	0.7533 ± 0.0145 ^c^
0.5	4.667 ± 0.088 ^d^	3.65 ± 0.05 ^bc^	0.247 ± 0.009 ^e^	2.94 ± 0.04 ^e^	0.0411 ± 0.0007 ^d^	0.7867 ± 0.020 ^c^
1	4.967 ± 0.088 ^d^	3.817 ± 0.91 ^b^	0.289 ± 0.013 ^d^	3.723 ± 0.069 ^d^	0.0441 ± 0.0012 ^c^	0.8467 ± 0.0176 ^b^
2.5	5.3 ± 0.115 ^c^	3.897 ± 0.41 ^ab^	0.338 ± 0.015 ^c^	4.283 ± 0.095 ^c^	0.047 ± 0.0009 ^b^	0.87 ± 0.0116 ^ab^
4	6.533 ± 0.145 ^a^	4.047 ± 0.384 ^ab^	0.488 ± 0.008 ^a^	5.313 ± 0.097 ^a^	0.0529 ± 0.0014 ^a^	0.91 ± 0.006 ^a^
5	5.633 ± 0.088 ^b^	4.243 ± 0.325 ^a^	0.451 ± 0.009 ^b^	4.653 ± 0.000 ^b^	0.0474 ± 0.0004 ^b^	0.8667 ± 0.012 ^ab^

Treatment data are represented as mean ± SE (*n* = 3). Treatment means with different letters indicate significant differences according to Duncan’s test (*p* < 0.05).

**Table 3:** Si concentration screening.

Si	Plant Height	Stem Diameter	Shoot		Root	
(mM)	(cm)	(mm)	Dry Weight (g)	Fresh Weight (g)	Dry Weight (g)	Fresh Weight (g)
Root application of Si						
0	3.63 ± 0.115 ^d^	3.83 ± 0.127 ^e^	0.16 ± 0.006 ^e^	1.98 ± 0.039 ^d^	0.017 ± 0.001 ^e^	0.53 ± 0.026 ^d^
0.25	3.83 ± 0.145 ^d^	3.88 ± 0.061 ^e^	0.18 ± 0.003 ^e^	2.06 ± 0.041 ^d^	0.0195 ± 0.000 ^e^	0.64 ± 0.025 ^c^
0.5	4.5 ± 0.153 ^c^	4.38 ± 0.096 ^c^	0.25 ± 0.022 ^d^	2.23 ± 0.036 ^d^	0.0245 ± 0.001 ^d^	0.79 ± 0.017 ^b^
0.75	5.53 ± 0.176 ^d^	4.68 ± 0.036 ^ab^	0.34 ± 0.01 ^bc^	3.30 ± 0.132 ^b^	0.0394 ± 0.001 ^c^	0.88 ± 0.017 ^b^
1	6.23 ± 0.145 ^a^	4.84 ± 0.032 ^a^	0.42 ± 0.009 ^a^	4.22 ± 0.087 ^a^	0.0485 ± 0.001 ^a^	1.036 ± 0.064 ^a^
1.5	4.533 ± 0.145 ^c^	4.50 ± 0.045 ^bc^	0.35 ± 0.019 ^b^	2.83 ± 0.1 ^c^	0.0419 ± 0.001 ^bc^	0.833 ± 0.026 ^b^
2	4.367 ± 0.12 ^c^	4.14 ± 0.045 ^d^	0.31 ± 0.009 ^c^	2.73 ± 0.151 ^c^	0.0432 ± 0.001 ^b^	0.583 ± 0.020 ^cd^
Foliar spray application of Si						
0	3.533 ± 0.115 ^c^	3.81 ± 0.096 ^c^	0.13 ± 0.013 ^e^	1.94 ± 0.056 ^e^	0.014 ± 0.001 ^e^	0.51 ± 0.012 ^d^
0.25	3.86 ± 0.145 ^c^	3.91 ± 0.027 ^c^	0.17 ± 0.008 ^d^	1.98 ± 0.087 ^e^	0.019 ± 0.00 ^d^	0.54 ± 0.012 ^d^
0.5	4.36 ± 0.067 ^b^	4.18 ± 0.115 ^b^	0.19 ± 0.003 ^d^	2.36 ± 0.116 ^d^	0.029 ± 0.001 ^c^	0.61 ± 0.009 ^c^
0.75	5.96 ± 0.067 ^a^	4.23 ± 0.078 ^b^	0.28 ± 0.023 ^c^	3.43 ± 0.098 ^c^	0.040 ± 0.001 ^b^	0.79 ± 0.017 ^b^
1	6.26 ± 0.088 ^a^	4.74 ± 0.033 ^a^	0.46 ± 0.021 ^a^	4.54 ± 0.057 ^a^	0.047 ± 0.001 ^a^	0.99 ± 0.032 ^a^
1.5	4.73 ± 0.12 ^b^	4.22 ± 0.018 ^b^	0.39 ± 0.006 ^b^	3.98 ± 0.078 ^b^	0.046 ± 0.002 ^a^	0.80 ± 0.028 ^b^
2	4.6 ± 0.153 ^b^	4.14 ± 0.052 ^b^	0.31 ± 0.013 ^c^	2.57 ± 0.056 ^d^	0.045 ± 0.002 ^a^	0.48 ± 0.020 ^d^

Treatment data are represented as mean ± SE (*n* = 3). Treatment means with different letters indicate significant differences according to Duncan’s test (*p* < 0.05).

**Table 4:** Primer sequences for real−time fluorescent quantitative PCR.

Gene	Gene ID	Primer Sequence (5′ to 3′)	Gene Function
*Rubisco*	Solyc07g066530	F: CGCTCAAACCTTAGTCACCCTCAAG	Rubisco auxiliary enzyme (rubisco)
		R: TGCCAGAACCATCTCACTCCTATCC	
*RCA*	Solyc10g086580	F: ATCGGATGACCAACAGGACATTGC	Ribulose diphosphate carboxylase/oxygenase activator enzyme (RCA)
		R: CTTGACCTTTGCCTCCCCATACAC	
*SBPcase*	Solyc05g052600	F: GGGATCACAGGAAGAGAGCAAGTTG	1, 7−diphosphate sedum heptanose−esterase (SBPcase)
		R: TCAAGAATCCTAACGGTGCCACTTC	
*TK*	Solyc10g018300	F: TTGGCTTGATCCCGTATTGTGCTAC	Transketoolase (TK)
		R: GCTCCTGCTGTCTCATTACCATCTG	
*Ru5PK*	Solyc08g076220	F: CCATGTACGATGAGCGTGTGAGAG	Ribulose 5−phosphate kinase (Ru5PK)
		R: TGAGTTGGGAGCACTTCAATGACTG	
*NADP−GAPDH*	Solyc12g094640	F: CAAAGGCTGTGTCTCTAGTGCTACC	3−phosphoglycerate dehydrogenase (NADP−GAPDH)
		R: CTTGACCATGTCGTCTCCCATAACC	
*ATP*	Solyc06g072540	F: GGCGACGGTTTGTTGATACAAGAAG	ATP synthase subunit (ATP)
		R: AAGAGGCTCATATACGGAACAACGC	
*Actin*	Solyc04g011500.3	F: TGGGTCAAAAAGACGCCTATG	
		R: ATAATCTGGGTCATCTTTTCACGA	

## Data Availability

Not applicable.
